# An Optimal Diagnostic Strategy for Tuberculosis in Hospitalized HIV-Infected Patients Using GeneXpert MTB/RIF and Alere Determine TB LAM Ag

**DOI:** 10.1128/JCM.01032-20

**Published:** 2020-09-22

**Authors:** Aliasgar Esmail, Anil Pooran, Natasha F. Sabur, Mohammed Fadul, Mantaj S. Brar, Suzette Oelofse, Michele Tomasicchio, Keertan Dheda

**Affiliations:** a Centre for Lung Infection and Immunity, Division of Pulmonology, Department of Medicine, University of Cape Town, Cape Town, South Africa; b UCT Lung Institute & South African MRC/UCT Centre for the Study of Antimicrobial Resistance, University of Cape Town, Cape Town, South Africa; c Department of Respirology, St. Michael’s Hospital, University of Toronto, Toronto, Canada; d Division of General Surgery, Mount Sinai Hospital, University of Toronto, Toronto, Canada; e Institute of Infectious Diseases and Molecular Medicine, University of Cape Town, Cape Town, South Africa; f Faculty of Infectious and Tropical Diseases, Department of Infection Biology, London School of Hygiene and Tropical Medicine, London, United Kingdom; Carter BloodCare and Baylor University Medical Center

**Keywords:** tuberculosis, HIV, lipoarabinomannan, GeneXpert MTB/RIF, *Mycobacterium tuberculosis*, human immunodeficiency virus

## Abstract

The diagnosis of tuberculosis (TB) in HIV-infected patients is challenging. Both a urinary lipoarabinomannan (LAM) test (Alere TB LAM) and GeneXpert-MTB/RIF (Xpert) are useful for the diagnosis of TB. However, how to optimally integrate Xpert and LAM tests into clinical practice algorithms remain unclear. We performed a *post hoc* analysis of 561 HIV-infected sputum-expectorating patients (median CD4 count of 130 cells/ml) from a previously published randomized controlled trial evaluating the LAM test in hospitalized HIV-infected patients with suspected TB. We evaluated 5 different diagnostic strategies using sputum culture as a reference standard (Xpert alone, LAM alone, sequential Xpert followed by LAM and vice versa [LAM in Xpert-negative patients and Xpert in LAM-negative patients], and both tests concurrently [LAM + Xpert]). A cost-consequence analysis was performed. Strategy-specific sensitivity and specificity, using culture as a reference, were similar with the Xpert-only and sequential and concurrent strategies. However, when any positive TB-specific test was used as a reference, the incremental yield of LAM over Xpert was 29.6% (45/152) and that of Xpert over LAM was 75% (84/11). The incremental yield of LAM increased with decreasing CD4 count. The costs per TB case diagnosed were similar for the sequential and concurrent strategies ($1,617 to $1,626). In sputum-expectorating hospitalized patients with advanced HIV and access to both tests, concurrent testing with Xpert and LAM may be the best strategy for diagnosing TB. These data inform clinical practice in settings where TB and HIV are endemic.

## Abstract (precis)

The diagnosis of tuberculosis (TB) in HIV-infected patients is challenging. Both a urinary lipoarabinomannan (LAM) test (Alere TB LAM) and GeneXpert-MTB/RIF (Xpert) are useful for the diagnosis of TB. However, how to optimally integrate Xpert and LAM tests into clinical practice algorithms remain unclear. We performed a *post hoc* analysis of 561 HIV-infected sputum-expectorating patients (median CD4 count of 130 cells/ml) from a previously published randomized controlled trial evaluating the LAM test in hospitalized HIV-infected patients with suspected TB.

## INTRODUCTION

In sub-Saharan Africa, the co-epidemics of tuberculosis (TB) and HIV are out of control (1). Approximately 70% of all incident TB cases in HIV-infected persons occur in Africa, and 17% of the total TB deaths are associated with HIV (2). The high case fatality rate from HIV-TB coinfection is related to disseminated and extrapulmonary forms of TB (EPTB) in patients with advanced immunosuppression (3), where the diagnosis is often missed or delayed. Indeed, postmortem studies demonstrate that TB is the cause of death in 40 to 60% of hospitalized HIV patients (4–6), with the diagnosis having been missed in over 45% of patients (4–6).

However, two tests have recently been shown to be effective for TB diagnosis in the setting of advanced HIV coinfection. The GeneXpert MTB/RIF test (Xpert) is an automated, sputum-based, real-time PCR assay that permits rapid identification of Mycobacterium tuberculosis and determination of rifampicin resistance (7). However, while access to Xpert testing has been proven to reduce time to treatment initiation (8, 9), it performs suboptimally in HIV-infected patients, who often have sputum smear-negative disease (10). The Alere Determine TB LAM Ag lateral flow strip test is a bedside urine-based test that identifies lipoarabinomannan (LAM), a glycolipid component of the mycobacterial cell wall, and provides a result in 25 min (11). It is World Health Organization (WHO) approved for use in HIV-infected patients with advanced immunosuppression (12, 13) and has been shown to reduce mortality in hospitalized HIV-positive patients when used to guide TB treatment initiation (14, 15). The urine LAM test has obvious advantages, as it is a point-of-care test facilitating rapid treatment initiation, the sample is easy to collect, and sample collection does not entail the same infection control concerns as that of sputum.

Current WHO guidelines recommend urine LAM testing in all HIV-infected hospitalized patients with advanced immunosuppression (CD4 count of <100 cells/ml or stage 3/4 disease) or who are seriously ill, irrespective of signs or symptoms of TB (16). Thus, the LAM test should be used as a screening test in this population. In hospitalized HIV-infected persons with a CD4 count of >100 cells/ml, LAM testing is also indicated in those with signs and symptoms of TB.

However, the guidelines are unclear on how newer tests like Xpert and the LAM test should be integrated into clinical practice in high-HIV-prevalence settings. This is a critical unanswered question in settings where TB and HIV are endemic and where, often, either only one or both tests are available. Thus, it is unclear whether, in individual test-specific settings, performing testing sequentially or concurrently may be more useful; the cost implications thereof remain unstudied. To address this question, we evaluated the test performance and cost consequence (per TB case diagnosed) of 5 testing strategies (individual [Xpert or urine LAM test alone], sequential [Xpert in LAM-negative patients or urine LAM test in Xpert-negative patients], or concurrent testing) in a large cohort of sputum-expectorating hospitalized HIV-infected patients that formed part of a larger parent study, the results of which are reported elsewhere (14).

## MATERIALS AND METHODS

### Study design, conduct, and oversight.

This study represents a *post hoc* subgroup analysis of data from a pragmatic, randomized, parallel-arm, multicenter study with stratified randomization by country. The primary analysis has already been reported (14). Prior to patient recruitment into the parent study, the study was approved by the appropriate national regulatory authorities, by the University of Cape Town Human Research Ethics Committee (HREC 007/2012), and by the ethics committee at each site. All patients provided informed consent in their first language prior to enrollment into the parent study. This trial was registered with Clinicaltrials.gov, number NCT01770730 (https://clinicaltrials.gov/ct2/show/NCT01770730).

### Study patients, enrollment criteria, and randomization.

Patients admitted to 10 urban or periurban hospitals in South Africa, Tanzania, Zambia, and Zimbabwe were screened for study inclusion. A detailed description of hospital care, HIV and TB prevalence, routine clinical care, and the diagnostics infrastructure at each hospital was reported previously (14). The inclusion criteria included (i) HIV infection; (ii) at least one of the following symptoms: current fever or cough, drenching night sweats, or self-reported loss of weight; (iii) illness severe enough to necessitate hospitalization; (iv) age of ≥18 years; (v) granting of informed consent. The exclusion criteria included (i) receipt of any anti-TB medication in the 60 days prior to testing and (ii) inability to provide at least 30 ml urine. Eligible patients were randomized to receive standard available TB diagnostics at each center or standard TB diagnostics plus adjunctive LAM, using centralized computer-generated allocation lists, stratified by country. The patients and the study team were not blinded to both allocation and test results.

### Procedures and standard clinical management.

In addition to a urine specimen, patients were asked to expectorate a minimum of two sputum samples for routine TB diagnosis. For patients unable to self-expectorate, sputum induction was employed. Clinical assessment by the attending physicians and chest X-ray facilities were available in most cases; however, additional radiology and nonsputum sampling were differentially available in study hospitals, and requesting these investigations was at the discretion of the attending clinical team. Xpert was performed on ∼1 ml of sputum, according to manufacturer’s instructions (Cepheid, Inc., USA), at all study sites. The urine LAM test was performed at the bedside, according to manufacturer’s instructions (Alere, USA). A grade 2 cutoff point or higher was deemed a positive urine LAM result. The attending clinical team made all decisions regarding patient therapy and initiation of anti-TB treatment and the timing thereof, including acting upon the LAM test results. The WHO guidelines for the treatment of smear-negative tuberculosis were routinely used at study hospitals.

### Patient selection for *post hoc* analysis.

**FIG 1:**
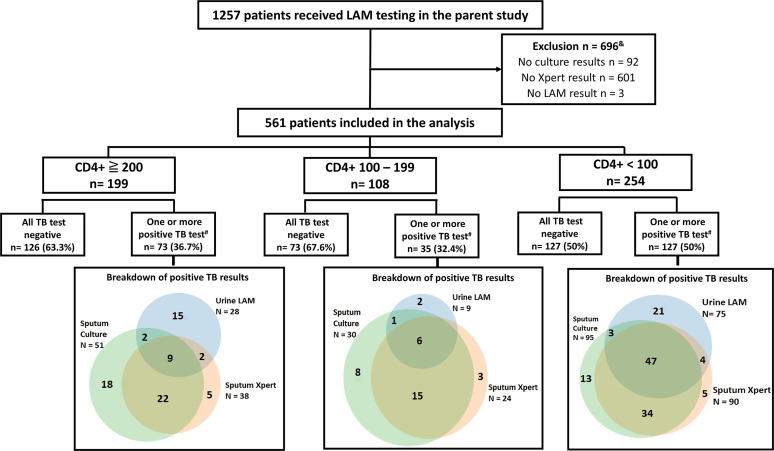
Flow diagram demonstrating patient selection and distribution of TB-positive results (LAM, lipoarabinomannan testing; Xpert, GeneXpert MTB/RIF). &, Excluded patients had similar demographic characteristics and approximately 27% were sputum scarce. #, LAM and/or Xpert and/or Bactec MGIT 960 TB culture-positive.

For this analysis, only patients randomized to the LAM test group and for whom Xpert and sputum culture results were available were included (561 patients of the 1,257 patients randomized to the LAM test group) (Fig. 1).

### Outcomes and statistical analysis.

The goal of this analysis was to assess the utility of single or sequential TB testing strategies incorporating urine LAM and/or Xpert in HIV-infected patients admitted for suspected TB. The sensitivity, specificity, positive predictive value (PPV), and negative predictive value (NPV) were calculated for 5 different strategies using sputum culture positivity as a reference standard: (i) performing Xpert alone; (ii) performing the urine LAM test alone; (iii) performing Xpert only in patients with an initial negative LAM result, (iv) performing LAM testing only in patients with an initial negative Xpert result, and (v) performing Xpert and LAM concurrently (LAM+Xpert).

In addition, the incremental yield of LAM and Xpert tests in the sequential testing strategies (Xpert in LAM-negative patients and LAM in Xpert-negative patients) was determined using either sputum culture positivity or any positive TB test (LAM, Xpert, and/or sputum culture) as a reference standard. An *a priori* subgroup analysis was performed on those with CD4 counts of ≤200 and ≤50 cells/mm^3^, as the diagnostic performance of urine LAM improves with lower CD4 counts (17). All analyses were performed using Stata V13 (StataCorp). Exact binomial 95% confidence intervals were calculated for proportions, and differences in proportions were calculated (with 95% confidence intervals using a normal approximation) for comparisons between diagnostic approaches.

### Economic evaluation (cost analysis).

A cost-consequence analysis was performed from the South African health care provider perspective to evaluate the different diagnostic strategies 1 to 5 mentioned above. Costs were expressed in 2018 U.S. dollars at an exchange rate of 13.20 South African rands to 1 U.S. dollar (http://wdi.worldbank.org/table/4.16). Costs were subsequently inflated to the year of analysis where appropriate using the World Bank Consumer Price Index for South Africa (https://data.worldbank.org/indicator/FP.CPI.TOTL?locations=ZA). The unit cost of Xpert was obtained from the National Health Laboratory Service (NHLS) and represents the actual cost incurred by the South African National TB Program. Such estimates have been used in other health economic studies (18–20). The unit cost of the urine LAM test was calculated using an ingredients approach and included the cost of the assay (provided by Alere), other consumables, and staff. The cost of anti-TB treatment for 6 months was obtained from the WHO South African tuberculosis finance profile (21). No discount rate was applied due to the short time frame of the analysis. Model probabilities were calculated based on test sensitivities and specificities reported in Table 2. Outcomes were normalized to 1,000 patients screened per strategy. The cost-effectiveness of each strategy was reported as the cost per culture-positive case diagnosed and started on treatment (per 1,000 patients screened). Other effectiveness measures were also considered (see the supplemental text). A univariate analysis was performed to determine the effect of varying specific parameter inputs for each strategy. Details of all costs, model parameters, and model assumptions are provided in the supplemental text.

## RESULTS

Of the 1,257 patients randomized to the LAM study arm, 561 patients were selected for analysis based on availability of sputum culture results and Xpert results for analysis (Fig. 1). The baseline patient characteristics are summarized in Table 1. The reference standard for the diagnosis of TB in the primary analysis was a positive sputum culture, which was reported for 31.7% of this cohort (178/561). Any positive TB-specific test (Xpert, urine LAM test, or sputum culture) was used as a reference standard in secondary analyses.

**TABLE 1:** Demographics and clinical characteristics of the study cohort

Variable	All patients (*n* = 561)	Patients stratified to CD4 counts			*P* value^*a*^
		≤200 mm^3^ (*n* = 361)	>200 mm^3^ (*n* = 143)	Unknown (*n* = 57)	
Median age, yrs (95% CI)	36.0 (18–75)	37.3 (19–70)	37.6 (18–75)	36.8 (19–61)	0.31
No. of males:females (% male)	294:267 (52.4)	190:171 (52.6)	77:66 (53.8)	27:30 (47.3)	0.71
Country of origin, no. (%)					0.79
South Africa	151	70 (27.5)	74 (29.1)	7 (13.5)	
Tanzania	193	96 (37.7)	97 (38.2)	0 (0.0)	
Zambia	217	89 (34.9)	83 (32.7)	45 (86.5)	
Median Karnofsky score (95% CI)	60 (20–90)	60.0 (20–90)	60.0 (30–90)	50.0 (50–60)	0.91
Median CD4 count, cells/mm^3^ (95% CI)					
Total	99.5 (1–933)	58 (1–199)	320 (201–933)		**0.0001**
Patients with any positive TB result	73.0 (1–695)	44 (1–199)	283 (206–695)		**0.0001**
Median BMI (95% CI)^*b*^	18.7 (9–37)	18.6 (9–27)	19.4 (10–37)	17.1 (16.6–19.3)	**0.002**
No. of positive culture results/total (%)	178/561 (31.7)	127/361 (35.2)	35/143 (24.5)	16/57 (28.1)	**0.02**

a Value for the group with CD4 counts of ≤200 versus those with count of >200 cells/mm^3^. Boldface indicates statistical significance.

b BMI, body mass index.

### Test characteristics of single, sequential, and concurrent testing strategies incorporating the LAM and Xpert tests in hospitalized patients irrespective of CD4 counts.

The sensitivities, specificities, PPVs, and NPVs (95% confidence intervals [CIs]) of the different strategies were as follows (Table 2): for Xpert only, 74.7% (67.7% to 80.9%), 95.1% (92.4% to 97.0%), 87.5% (81.2% to 92.3%), and 89.0% (85.6% to 91.9%), respectively; for LAM testing only, 38.2% (30.9% to 45.7%), 88.3% (84.6% to 91.3%), 60.2% (50.5% to 69.3%), and 75.5% (71.3% to 79.4%), respectively; and for sequential (Xpert in LAM-negative patients and LAM in Xpert-negative patients) and concurrent (LAM+Xpert) testing, 78.1% (71.3% to 83.9%), 85.1% (81.2% to 88.5%), 70.9% (65.5% to 75.8%) and 89.3% (86.3% to 91.7%), respectively. When the sequential/concurrent testing strategies were compared to Xpert only, there was a significant decrease in specificity (10.0% difference [95% CI, 6.2% to 14.6%]; *P* < 0.0001) and PPV (16.6% difference [95% CI, 9.1% to 25.7%]; *P* = 0.001). However, no difference in sensitivity (−3.4% difference [95% CI, −12.2% to 5.4%]; *P* = 0.22) and NPV was observed (−0.3% difference [95% CI, −4.6% to 4.0%]; *P* = 0.45).

**TABLE 2:** Diagnostic performance of sputum-based Xpert MTB/RIF and urine-based Alere Determine TB LAM Ag testing irrespective of CD4 count^*a*^

Test strategy	% (95% CI)				Likelihood ratio	
	Sensitivity	Specificity	PPV	NPV	Positive	Negative
Xpert MTB/RIF only	74.7 (67.7–80.9)	95.1 (92.4–97.0)	87.5 (81.2–92.3)	89.0 (85.6–91.9)	15.1 (9.7–23.6)	0.27 (0.2–0.34)
Urine LAM test only	38.2 (31.0–45.7)	88.3 (84.6–91.3)	60.2 (50.5–69.3)	75.5 (71.3–79.4)	3.3 (2.3–4.5)	0.7 (0.6–0.8)
Sequential or concurrent testing^*b*^	78.1 (71.3–83.9)	85.1 (81.2–88.5)	70.9 (65.5–75.8)	89.3 (86.3–91.7)	5.3 (4.1–6.8)	0.3 (0.2–0.34)

a Diagnostic performance of sputum-based Xpert MTB/RIF and urine-based Alere Determine TB LAM Ag testing using single and sequential testing strategies in HIV-infected hospitalized patients irrespective of CD4 count and using sputum culture positivity as the reference standard.

b Sequential testing refers to performing Xpert for LAM-negative patients or LAM for Xpert-negative patients; concurrent testing refers to both tests being performed at the same time rather than reflex testing based on the initial test result (in all 3 scenarios, the number of TB cases diagnosed remains the same, and hence, sensitivities and specificities are identical).

### Test characteristics of single and sequential/concurrent testing strategies incorporating LAM and Xpert testing in hospitalized patients with CD4 counts of ≤50 and ≤200 cells/mm^3^.

When test performance was assessed in patients with CD4 counts of ≤50 cells/mm^3^, the LAM test-only strategy showed improved sensitivity and PPV (60.0% [47.1% to 72.0%] and 67.2% [53.7% to 79.0%], respectively) (Table 3) but a slightly reduced specificity and NPV (81.7% [72.9% to 88.6%] and 77.0% [68.1% to 84.4%], respectively), compared to the entire cohort (not stratified by CD4 count). A similar pattern was observed in the Xpert-only strategy, with a sensitivity, specificity, PPV, and NPV of 81.5% (71.3% to 89.2%), 94.3% (88.1% to 97.9%), 89.2% (79.8% to 95.2%), and 89.9% (83.8% to 94.2%), respectively (Table 3). However, sequential/concurrent testing did not show a significant difference in sensitivity compared to Xpert only (89.2% [79.1% to 95.6%]; *P* = 0.28) in this patient group. A similar pattern, albeit to a lesser extent, was observed when patients with CD4 counts of ≤200 cells/mm^3^ were analyzed (Table 3).

**TABLE 3:** Diagnostic performance of sputum-based Xpert MTB/RIF and urine-based Alere LAM testing in patients with CD4 counts of ≤50 and ≤200 cells/mm^3^^*a*^

Reference standard	CD4 count (cells/mm^3^)	Test strategy^*b*^	% (95% CI)			
			Sensitivity	Specificity	PPV	NPV
Sputum culture positivity	≤50	Xpert MTB/RIF only	81.5 (71.3–89.2)	94.3 (89.1–97.5)	89.2 (79.8–95.2)	89.9 (83.8–94.2)
		Urine LAM test only	60.0 (47.1–72.0)	81.7 (72.9–88.6)	67.2 (53.7–79.0)	76.6 (67.6–84.1)
		Sequential or concurrent testing	89.2 (79.1–95.6)	79.8 (70.8–87.0)	73.4 (62.3–82.7)	92.2 (84.6–96.8)
	≤200	Xpert MTB/RIF Only	80.3 (72.3–86.8)	94.9 (91.3–97.3)	89.5 (82.3–94.4)	90.0 (85.5–92.4)
		Urine LAM test only	44.9 (36.1–54.0)	88.1 (83.3–92.0)	67.1 (56.0–76.9)	74.8 (69.3–79.8)
		Sequential or concurrent testing	83.5 (75.8–89.5)	84.7 (79.5–89.1)	74.6 (66.7–81.6)	90.5 (85.8–94.0)

Any microbiological test positivity^*c*^	≤50	Xpert MTB/RIF only	71.3 (60.6–80.5)			77.1 (70.7–82.4)
		Urine LAM test only	66.7 (55.8–76.4)			74.3 (68.3–79.6)
		Sequential or concurrent testing	92.0 (84.1–96.7)			92.3 (85.5–96.1)
	≤200	Xpert MTB/RIF only	69.9 (62.3–76.9)			80.3 (76.4–83.8)
		Urine LAM test only	52.2 (44.1–60.0)			71.9 (68.6–75.1)
		Sequential or concurrent testing	87.1 (81.0–91.8)			90.5 (86.5–93.4)

a Diagnostic performance of sputum-based Xpert MTB/RIF and urine-based Alere LAM in single, sequential, and concurrent testing strategies in HIV-infected hospitalized patients with CD4 counts of ≤50 and ≤200 cells/mm^3^ using sputum culture or any microbiological test positivity as a reference.

b Sequential testing refers to performing Xpert in LAM-negative patients or LAM in Xpert-negative patients; concurrent testing refers to performing both Xpert and LAM concurrently.

c Specificity and PPV could not be determined when any microbiological test positivity is used as a reference standard (as this will always equate to 100%).

### Incremental yield of urine LAM testing and Xpert in sequential testing strategies.

**FIG 2:**
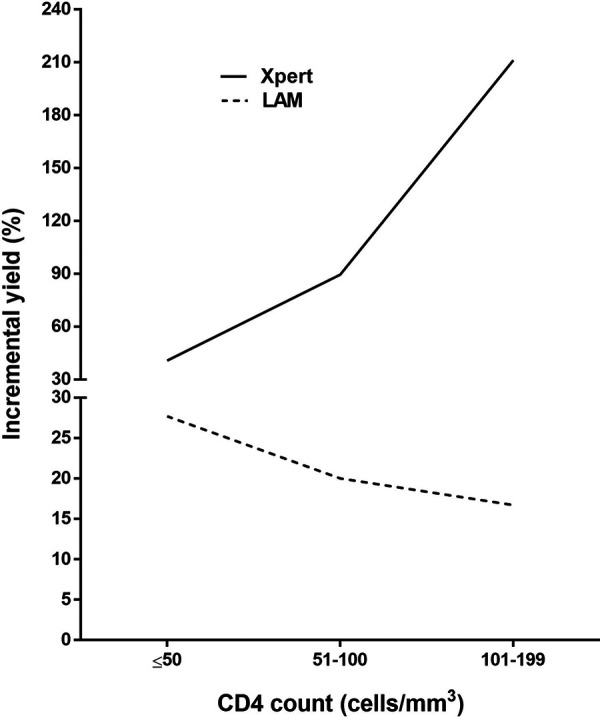
Incremental yield of LAM testing (LAM positivity in Xpert-negative patients) and Xpert (Xpert positivity in LAM-negative patients) when any positive TB specific test was used as a reference standard in patients with CD4 counts of ≤200 cells/mm^3^.

The incremental yield of urine LAM testing in the strategy of using the LAM test in Xpert-negative patients was 4.5% (6 urine LAM-positive results/132 Xpert-positive results) (Table 4), while the incremental yield of Xpert testing in the LAM-negative strategy was 104% (71 Xpert-positive results/68 urine LAM-positive results). When the analysis was repeated using any positive TB microbiological test (urine LAM test, Xpert, or sputum culture) as a reference standard for the diagnosis of TB (as is the case in clinical practice), the incremental yield of LAM in the strategy of using the LAM test in Xpert-negative patients increased from 4.5% (with sputum culture as a reference) to 29.6% (Table 4). The incremental yield of Xpert or LAM testing, using any positive microbiological test for TB as a reference standard, was also dependent on CD4 count (Fig. 2).

**TABLE 4:** Incremental yield of urine LAM and Xpert in sequential testing strategies using different reference standards

Testing strategy	Diagnostic reference standard^*a*^	% of patients diagnosed with TB with the initial test^*b*^	Incremental diagnostic yield (%) in patients who test negative with the initial test^*c*^
Xpert followed by LAM in Xpert-negative patients	TB culture	74.7 (133/178)	4.5 (6/133)
	Any TB-positive test	64.7 (152/235)	29.6 (45/152)
LAM followed by Xpert in LAM-negative patients	TB culture	38.2 (68/178)	104 (71/68)
	Any TB-positive test	47.6 (112/235)	75 (84/112)

a Any positive test refers to a positive result obtained from MGIT sputum culture, Xpert MTB/RIF, or urine LAM testing. It is acknowledged that inclusion of Xpert or LAM in the reference standard represents inclusion bias that tends to overestimate sensitivity (data are provided for purposes of comparison).

b Numbers in parentheses are number of positive patients/total.

c Numbers in parentheses are Xpert-positive results/LAM-positive results.

### Economic evaluation.

The total costs of the sequential (LAM in Xpert-negative and Xpert in LAM-negative patients) and concurrent (LAM+Xpert) testing strategies were similar, ranging from $400,656 to $402,909 per 1,000 patients screened (Table 5). Costs associated with unnecessary treatment were higher in the sequential and concurrent testing strategies ($186,228 to $187,235 per 1,000 patients screened), as more individuals with false-positive test results were started on treatment. LAM testing only was the cheapest strategy, followed by Xpert only ($305,927 and $355,358 per 1,000 patients screened, respectively). The higher cost of sequential/concurrent testing was a consequence of more individuals being correctly diagnosed and subsequently started on treatment (248 patients in each strategy) compared to LAM only (121 patients) and Xpert only (237 patients). However, sequential/concurrent testing also had the fewest missed culture-positive TB cases (49 patients in each strategy) and empirically treated patients (195 patients in each strategy; see Table S6 in the supplemental material). The costs per culture-positive case diagnosed and started on treatment for the sequential and concurrent testing strategies were very similar (costs for LAM in Xpert-negative patients, Xpert in LAM-negative patients, and LAM+Xpert strategies were $1,617, $1,621, and $1,625, respectively) but still slightly higher than for Xpert only ($1,500). LAM testing only remained the least cost-effective strategy ($2,525) (Table 5).

**TABLE 5:** Costs, outcomes, and cost-effectiveness for different test strategies^*a*^

Strategy and test	Cost/1,000 patients screened			No. of patients diagnosed/1,000 screened^*b*^	Cost-effectiveness	
	Total	Diagnostic	Treatment		Cost per patient diagnosed^*c*^	Cost difference (compared to Xpert only)
Single						
Xpert	355,358 (353,256–359,086)	14,375 (14,375–14,375)	340,983 (338,881–344,711)	237.1 (214.9–256.7)	1,500 (1,399–1,645)	
LAM	305,927 (305,271–307,949)	3,557 (3,557–3,557)	302,370 (303,714–304,392)	121.3 (98.4,145.4)	2,525 (2,120–3,124)	1,025
Sequential						
LAM in Xpert-negative patients	400,656 (412,366–406,317)	15,049 (14,997–15,014)	385,608 (397,370–391,303)	247.9 (205.7–288.6)	1,617 (1,429–1,976)	117
Xpert in LAM-negative patients	401,476 (405,139–414,150)	16,978 (16,954–16,991)	384,499 (388,185–397,160)	247.7 (219–278.1)	1,621 (1,458–1,892)	122
Concurrent (Xpert+LAM)	402,909 (400,992–406,859)	17,932 17,932–17,932)	384,977 (383,061–388,927)	247.9 (226.3–266.3)	1,626 (1,507–1,799)	127

a Costs, outcomes, and cost-effectiveness for single, sequential, and concurrent test strategies to diagnose TB in hospitalized patients with advanced HIV using Xpert MTB/RIF and LAM urine tests. Costs are expressed in 2018 U.S. dollars, with 95% CIs in parentheses.

b Number of culture-positive patients correctly diagnosed and started on treatment per 1,000 patients screened, with 95% CI in parentheses.

c Cost per culture-positive patient diagnosed and started on treatment.

A univariate sensitivity analysis indicated that TB prevalence, the rate of empirical treatment, and TB treatment costs were the most influential parameters on cost-effectiveness (Fig. S3). Subsequently, the cost per culture-positive patient diagnosed and started on treatment for each strategy was plotted against increasing TB prevalence and empirical TB treatment rate, as these tend to vary widely in different settings. The cost-effectiveness values decreased as prevalence increased, but the rankings remained unchanged (Xpert alone was cheapest, followed by sequential/concurrent strategies). However, the magnitude of the cost difference between the sequential/concurrent testing strategies and Xpert only decreased (Fig. S4A). A similar reduction in the cost difference between strategies was observed when empirical treatment rate increased, until ∼70%, when the cost per outcome of the sequential/concurrent strategies became less than that of Xpert alone (Fig. S4B). In both cases, the LAM-only strategy was the least cost-effective.

## DISCUSSION

We evaluated the benefit of using Xpert and urine LAM testing performed either singly, sequentially, or concurrently in sputum-expectorating, hospitalized, HIV-infected patients with suspected TB. Our key findings were as follows: (i) Xpert performs better than the urine LAM test, irrespective of CD4 count; (ii) serial/concurrent testing using Xpert and LAM (Xpert in LAM-negative patients, LAM in Xpert-negative patients, and LAM+Xpert) improves sensitivity over either test alone; (iii) LAM+Xpert may be the most appropriate strategy for diagnosing TB, especially in the setting of advanced immunosuppression (CD4 count of ≤200 cells/mm^3^), since each of these tests independently diagnoses patients with active TB, and moreover, the incremental yield of LAM over Xpert increases with increasing immunosuppression; and (iv) the costs per TB case diagnosed and started on treatment were similar for the sequential and concurrent strategies ($1,617 to $1,625) and only ∼$120 more than the Xpert-only strategy.

There are few data about how to optimally use or combine different TB tests in a clinical setting. A positive result with either Xpert or LAM is considered diagnostic for TB and will prompt the clinician to treat for TB. Given this consideration, we analyzed the data using 2 reference standards: (i) sputum culture positivity and (ii) any TB microbiological test positivity. Using the former (Table 4), urine LAM provided a modest incremental yield of 4.5% (6 patients) in Xpert-negative patients. In contrast, when Xpert, sputum culture, or LAM positivity (i.e., any microbiological test positivity) was used as a reference, the urine LAM test incremental yield was ∼30% in Xpert-negative patients. This group (Xpert negative and LAM positive) had the lowest Karnofsky scores and highest mortality in our cohort (Table S2). When only patients with CD4^+^ counts of ≤50 cells/μl were included, LAM sensitivity improved by 20% and 26% when sputum culture and any positive microbiological test were used as references, respectively. This suggests that the patients with lower CD4 counts benefit most from LAM testing. However, even in this group with advanced HIV, the overall sensitivity of Xpert was superior to that of LAM (Table 3). Thus, our data support the need for a strategy incorporating both tests, especially at lower CD4 counts (≤200 cells/mm^3^), given that each test independently identifies patients with active TB (in this scenario, a patient’s urine and sputum samples would be taken simultaneously and both tests would be run concurrently).

Our findings are consistent with a Ugandan study in which a combination of LAM and sputum Xpert testing had a greater sensitivity than either test alone (22). Importantly, the parent study (17) demonstrated a survival benefit with a LAM-guided testing strategy due to more rapid treatment initiation. More recently, the STAMP trial, which assessed patient outcomes using a concurrent LAM and Xpert diagnostic strategy, demonstrated significantly reduced mortality (7.1%) in patients with CD4 counts of <100 cells/μl and detected more TB cases compared to Xpert alone (21.9% versus 14.9%; *P* < 0.001) (15). Thus, in sputum-expectorating patients, concurrent testing leverages the advantages of both tests, i.e., LAM allows rapid treatment initiation while Xpert results are awaited to ascertain resistance to rifampicin (Xpert results may take up to 10 days in some settings where these diseases are endemic and has obvious relevance in settings where rifampicin resistance is common) (23, 24); this strategy is also broadly cost neutral. However, there is also a reduction in specificity with sequential/concurrent testing, especially at low CD4 counts, that subsequently results in a higher number of false positives. As such, the consequences of initiating treatment unnecessarily must also be considered when a testing strategy is chosen, especially when there is an increased risk of adverse drug reactions, such as those associated with second-line TB treatment. Furthermore, in patients who are unable to expectorate sputum, LAM testing is the obvious first choice, and our findings in this subgroup (patients with scarce sputum) have been reported separately (25).

There are no cost-related data to inform test selection in settings where TB is endemic. Our cost-consequence analysis indicated that the costs per culture-positive patient diagnosed and started on treatment for the sequential/concurrent strategies were similar ($1,617 to $1,626) and only marginally higher than that of using Xpert alone ($1,500). The cost similarity is explained by the LAM assay having a low unit cost, and it had little contribution to the overall strategy-specific costs. Treatment costs were greater with sequential/concurrent testing because more culture-positive patients were started on treatment than with the Xpert-only (11 more patients) and LAM test-only (127 more patients) strategies. However, the additional cost of ∼$120 for every TB patient diagnosed and started on treatment when LAM testing is incorporated into Xpert-based diagnostic strategies is likely justifiable, as hospitalized patients with advanced HIV immunosuppression are at higher risk for misdiagnosis and poor outcomes if treatment is not rapidly initiated (26). This is in agreement with other cost-effectiveness studies evaluating the addition of LAM testing to existing TB diagnostic tests (27), including one related to the STAMP trial (28). Implementation costs of these strategies would also likely be affordable given the relatively low LAM unit test cost and lack of need for additional infrastructure or expertise. Furthermore, the sequential/concurrent strategies were more cost-effective than Xpert only at high TB prevalence and high empirical treatment rates, making this a particularly economically attractive approach in such settings.

Our study has several limitations. We confined our analysis to patients who could expectorate sputum, yet LAM may be most beneficial in those whose sputum is scarce or who have EPTB. However, the main aim of our study was to inform diagnostic algorithms in settings where other rapid sputum-based diagnostics, such as Xpert, were available, and the findings in patients with scarce sputum were published elsewhere (25). Furthermore, this was a *post hoc* analysis, utilizing a limited subset of the patients recruited into the parent study, which may limit generalizability of the findings. However, patients were recruited from 4 different countries in sub-Saharan Africa, and this is the largest study to date comparing LAM test and Xpert incremental yields in patients from whom sputum could be obtained. Third, the parent study was conducted before the availability of Xpert Ultra, which is a more sensitive test (29), but it is unlikely that this would have impacted our conclusions (Ultra overall has an ∼5% increased sensitivity and a disadvantage of a higher false-positive rate in patients with a history of TB) (29). It is possible that the LAM test may provide additional diagnostic value when Xpert Ultra trace results in high-risk populations are interpreted (but not for detection of rifampicin resistance, which cannot be inferred using a trace readout). Finally, cost-effectiveness was calculated using clinical outcomes rather than single-utility metrics such as disability-adjusted life years (DALYs), so we cannot explicitly state that these strategies are cost-effective compared to Xpert only in the traditional sense, i.e., below the classical willingness-to-pay threshold of three times the per-capita GDP per DALY averted. Furthermore, we did not incorporate other clinical outcomes, such as death or additional TB cases missed due to insufficient data. Given the high mortality risk in this population, it is likely that inclusion of these effects would, if anything, have improved the cost-effectiveness of the sequential/concurrent strategies.

### Conclusion.

In sputum-expectorating hospitalized patients with advanced HIV and access to both tests, concurrent testing with Xpert and LAM is the most appropriate strategy for diagnosing TB. These data inform clinical practice in settings where TB and HIV are endemic.

## Supplementary Material

Supplemental file 1
