# Supplementary material for: An Optimal Diagnostic Strategy for Tuberculosis in Hospitalized HIV-Infected Patients Using GeneXpert MTB/RIF and Alere Determine TB LAM Ag
Source: J Clin Microbiol. 2020 Sep 22;58(10):e01032-20. doi: 10.1128/JCM.01032-20 (PMC7512177; doi:10.1128/JCM.01032-20)
Supplement: Supplemental file 1 [file JCM.01032-20-s0001.pdf]

SUPPLEMENTARY MATERIAL

An optimal diagnostic strategy for tuberculosis in hospitalized HIV-Infected patients using GeneXpert MTB/RIF and Alere Determine LAM

Aliasgar Esmail[a,b], Anil Pooran[a,b], Natasha F Sabur[a,c], Mohammed Fadul[a,b], Mantaj S Brar[d], Suzette Oelofse[a,b], Michele Tomasicchio[a,b], Keertan Dheda[a,b,e,f]#

[a]Centre for Lung Infection and Immunity, Division of Pulmonology, Department of Medicine, University of Cape Town, Cape Town, South Africa

[b]UCT Lung Institute & South African MRC/UCT Centre for the Study of Antimicrobial Resistance, University of Cape Town, Cape Town, South Africa

[c]Department of Respirology, St. Michael's Hospital, University of Toronto, Toronto, Canada

[d]Division of General Surgery, Mount Sinai Hospital, University of Toronto, Toronto, Canada

[e]Institute of Infectious Diseases and Molecular Medicine, University of Cape Town, Cape Town, South Africa

[f]Faculty of Infectious and Tropical Diseases, Department of Infection Biology, London School of Hygiene and Tropical Medicine, London, UK

KEYWORDS, tuberculosis, HIV, lipoarabinomannan, GeneXpert MTB/RIF

# Address correspondence to Keertan Dheda, keertan.dheda@uct.ac.za

MATERIALS AND METHODS

Economic analysis

A cost-effectiveness analysis was performed from the South African healthcare provider perspective to evaluate and compare the following strategies for diagnosing tuberculosis (TB) in hospitalized patients with advanced HIV suspected of TB: (i) performing Xpert alone (Xpert only); (ii) performing urine LAM alone (LAM only); (iii) performing Xpert only in patients with an initial Xpert negative result (Xpert in LAM-ve); (iv) performing urine LAM only in patients with an initial negative Xpert result (LAM in Xpert-ve); (v) performing Xpert and LAM concurrently (LAM+Xpert). Calculations were performed using Microsoft Excel (Microsoft) and GraphPad Prism 6.0 (GraphPad).

Costs

Costs were expressed in US$2018 at an exchange rate of ZAR13.20 to US$1 (http://wdi.worldbank.org/table/4.16). Costs were subsequently inflated to the year of analysis where appropriate using the World Bank Consumer Price Index for South Africa (https://data.worldbank.org/indicator/FP.CPI.TOTL?locations=ZA). No discount rate was applied due to the short timeframe of the analysis. The unit cost of Xpert was obtained from the National Health Laboratory Service (NHLS). The NHLS is a reference lab which provides services for the public healthcare system in South Africa, so these costs represent the actual costs incurred by the South African National TB Program. Such estimates have been used in other health economic studies (1-3). The unit cost of the urine LAM test was provided by Alere, the test suppliers of the Determine^{TM} TB LAM Ag lateral flow strip test

and included laboratory consumables and staff time. The cost of anti-TB treatment for 6 months in South Africa was estimated from the per patient TB budget as reported in the WHO South African tuberculosis finance profile (4). (Table S5).

Outcomes

Model probabilities were calculated based on test sensitivities and specificities reported in Table 2 of the main manuscript. TB prevalence for this specific population was similar to the prevalence reported in the main trial (5). The probabilities of a positive, negative, true positive, false positive, true negative and false negative test were subsequently calculated and normalised to 1000 patients screened per strategy.

Cost-effectiveness

Cost-effectiveness was expressed as the cost per culture positive case diagnosed and initiated on treatment (per 1000 patients screened) for each strategy.

Sensitivity analysis

A univariate sensitivity analysis was performed where a single parameter was changed to determine its effect on the cost per culture-positive patient diagnosed and initiated on TB treatment. Input values for probability estimates were varied based on clinical advice and on estimates from the literature. Cost estimates were either halved or doubled for the low and high input values, respectively.

Assumptions

The following assumptions were made for the analysis: (i) sputum culture was used as the gold standard TB diagnostic test. However, culture was not included in the costs as all patients were subjected to sputum culture in each strategy; (ii) any patient with a positive test in each strategy (Xpert or urine LAM) was assumed to be immediately initiated on treatment, which is in line with current clinical practice even without the availability of sputum culture; (iii) all patients initiating treatment based on a positive test result will complete a full 6-month course of first line anti-TB therapy; (iv) we assumed 30% of patients with a negative test result (LAM and/or Xpert) will be empirically initiated on treatment. Furthermore, based on clinical advice, we assumed 70% of empirically treated patients will complete a full 6-month course of anti-TB treatment whereas 30% will complete a 3-month course; (v) although Xpert MTB/RIF can detect rifampicin resistance, we did not incorporate the costs or outcomes associated with drug resistant TB into the analysis; (vi) additional diagnostic tests e.g. chest-x-ray and drugs, including those associated with HIV, were not included as they were assumed to be equivalent in each of the strategies; (vii) treatment outcomes (cure, died, etc) were not included in the outcomes due to insufficient data.

REFERENCES

1. Meyer-Rath G, Schnippel K, Long L, MacLeod W, Sanne I, Stevens W, Pillay S, Pillay Y, Rosen S. 2012. The Impact and Cost of Scaling up GeneXpert MTB/RIF in South Africa. PLoS ONE 7:e36966.
2. Pooran A, Pieterson E, Davids M, Theron G, Dheda K. 2013. What is the cost of diagnosis and management of drug resistant tuberculosis in South Africa? PLoS ONE 8:e54587.
3. Cleary SM, McIntyre D, Boulle AM. 2006. The cost-effectiveness of antiretroviral treatment in Khayelitsha, South Africa--a primary data analysis. Cost Eff Resour Alloc 4:20.
4. WHO. 2015. South Africa: Tuberculosis finance profile World Health Organization, Geneva, Switzerland. http://www.who.int/tb/country/data/profiles/en/index.html.
5. Peter JG, Zijenah LS, Chanda D, Clowes P, Lesosky M, Gina P, Mehta N, Calligaro G, Lombard CJ, Kadzirange G, Bandason T, Chansa A, Liusha N, Mangu C, Mtafya B, Msila H, Rachow A, Hoelscher M, Mwaba P, Theron G, Dheda K. 2016. Effect on mortality of point-of-care, urine-based lipoarabinomannan testing to guide tuberculosis treatment initiation in HIV-positive hospital inpatients: a pragmatic, parallel-group, multicountry, open-label, randomised controlled trial. Lancet 387:1187-97.

TABLES

Table S1: Diagnostic performance of sputum-based Xpert MTB/RIF and urine-based Alere Determine TB LAM Ag testing in single and sequential testing strategies in hospitalized HIV-infected patients (stratified by CD4 count) using sputum culture positivity as the reference standard (PPV - positive predictive value; NPV – negative predictive value)

**CD4 counts stratification**	**Test strategy**	**Sensitivity % (95% CI)**	**Specificity % (95% CI)**	**PPV % (95% CI)**	**NPV % (95% CI)**
CD4 count >200 cells/mm^3	Xpert MTB/RIF Only	60.0 (42.1 – 30.3)	96.4 (91.0 – 99.0)	84.0 (81.3 – 93.5)	88.4 (81.3 – 93.5)
	Urine LAM only	14.3 (4.8 – 52.6)	90.1 (83.0 – 94.9)	31.3 (11.0 – 58.7)	76.9 (68.7 – 83.9)
	*Sequential/concurrent testing using Xpert MTB/RIF and LAM	62.9 (44.9 – 78.5)	86.5 (78.7 – 92.2)	59.5 (42.1 - 75.2)	88.1 (80.5 – 93.5)
CD4 count ≤200 cells/mm^3	Xpert MTB/RIF Only	80.3 (72.3 – 86.8)	94.9 (91.3 – 97.3)	89.5 (82.3 – 94.4)	90.0 (85.5 – 92.4)
	Urine LAM only	44.9 (36.1 – 54.0)	88.1 (83.3 – 92.0)	67.1 (56.0 – 76.9)	74.8 (69.3 – 79.8)
	*Sequential/concurrent using Xpert MTB/RIF and LAM	83.5 (75.8 – 89.5)	84.7 (79.5 – 89.1)	74.6 (66.7 - 81.6)	90.5 (85.8 – 94.0)
CD4 count ≤100 cells/mm^3	Xpert MTB/RIF Only	83.5 (74.6 – 90.3)	94.3 (89.4 – 97.3)	90.0 (81.9 – 95.3)	90.2 (84.6 –94.3)
	Urine LAM only	51.5 (41.2 – 61.8)	84.1 (77.4 – 89.4)	66.7 (54.8 – 77.1)	73.7 (66.7 - 80.0)
	*Sequential/concurrent testing using Xpert MTB/RIF and LAM	86.6 (78.2 – 92.7)	80.9 (73.9 – 86.7)	73.7 (64.6 - 81.5)	90.7 (84.6 - 95.0)
	Xpert MTB/RIF Only	81.5 (71.3 – 89.2)	94.3 (89.1 – 97.5)	89.2	89.9

				(79.8 – 95.2)	(83.8 – 94.2)
CD4 count ≤50 cells/mm^3	Urine LAM only	60.0 (47.1 – 72.0)	81.7 (72.9 – 88.6)	67.2 (53.7 – 79.0)	76.6 (67.6 – 84.1)
	*Sequential/concurrent testing using Xpert MTB/RIF and LAM	89.2 (79.1 – 95.6)	79.8 (70.8 – 87.0)	73.4 (62.3 - 82.7)	92.2 (84.6– 96.8)

* Sequential testing refers to performing Xpert in LAM negative patients (Xpert in LAM-ve) or LAM in Xpert negative patients (LAM in Xpert -ve). Concurrent testing refers to performing LAM and Xpert concurrently (LAM+Xpert)

Table S2: Indicators of disease severity in HIV-infected patients suspected of TB according to LAM and Xpert test result.

	**LAM Pos and Xpert Neg (n = 45)**	**LAM Pos and Xpert Pos (n = 68)**	**LAM Neg and Xpert Pos (n = 84)**	**LAM Neg and Xpert Neg (n = 39)**
Median CD4 count; cells/mm^3 (95% CI)	63.5 (30.3, 182.9)	27.0 (20.3, 40.4)	98 (68.8, 130.9)	139 (61.7, 189.1)
Median Karnofsky score (95% CI)	50 (50, 60)	60 (50, 60)	60 (50, 70)	60 (50, 70)
Mortality; n (%)	12 (28.6%) (n = 42)	14 (22.2%) (n = 63)	15 (19.2%) (n= 78)	4 (10.8%) (n = 37)
Median Weight; kg (95% CI)	48 (45.1, 53.6) (n = 41)	48.0 (46.1, 50.0) (n = 65)	50.0 (46.8, 51.9) (n = 77)	50 (48.7, 55.0) (n= 31)

Table S3: Incremental yield of LAM over Xpert (LAM positive in patients who tested negative with Xpert) stratified according to CD4

CD4 grouped	Incremental yield using any positive test as reference %		Incremental yield using TB culture as a reference %	
	Total (n)	% incremental yield	Total (n)	% incremental yield
≤ 50	90	27.7 (18/65)	68	3.3 (2/59)
51 ≤ 100	43	20.0 (6/30)	35	3.7 (1/27)
101 ≤ 200	37	16.7 (4/24)	31	4.8 (1/21)
200 ≤ 500	48	65.0 (13/20)	34	11.8 (2/17)
> 500	8	25 (2/8)	6	16.6 (1/8)

Table S4: Incremental yield of Xpert over LAM (Xpert positive in patients who tested negative with LAM) stratified according to CD4

CD4 grouped	Incremental yield using any positive test as reference %		Incremental yield using TB culture as a reference %	
	Total (n)	% incremental yield	Total (n)	% incremental yield
≤ 50	90	40.7 (24/59)	68	52.5 (21/40)
51 ≤ 100	43	89.5 (17/19)	35	115.4 (15/13)
101 ≤ 200	37	211.1 (19/9)	31	266.6 (16/6)
200 ≤ 500	48	106.2 (17/16)	34	260.0 (11/4)
> 500	8	250.0 (5/2)	6	200.0 (4/2)

Table S5: Probability estimates and costs used in the cost-consequence analysis

**Probablility estimates**	**Value**	**Source**
Prevalence of TB in study population	0.317	[5]
Prevalence of TB in Xpert MTB/RIF negative patients	0.110	calculated
Prevalence of TB in LAM negative patients	0.245	calculated
Xpert MTB/RIF sensitivity	0.747	Table 2 of main manuscript
Xpert MTB/RIF sensitivity in urine LAM -ve patients	0.646	calculated
Xpert MTB/RIF specificity	0.951	Table 2 of main manuscript
Xpert MTB/RIF specificity in urine LAM -ve patients	0.962	calculated
Urine LAM sensitivity	0.382	Table 2 of main manuscript
Urine LAM sensitivity in Xpert -ve patients	0.133	calculated
Urine LAM specificity	0.882	Table 2 of main manuscript
Urine LAM specificity in Xpert -ve patients	0.896	calculated
TB treatment initiated if test (urine LAM or Xpert MTB/RIF) positive	1.00	assumption
TB treatment initiated if test negative (empirical treatment)	0.30	assumption
**Cost estimates**	**Value**	**Source**
Xpert MTB/RIF	$14.38	National Health Laboratory Service
Urine LAM	$3.56	Alere
6month course of DOTS-based TB treatment	$746.95	[4]
cost of those initiated on empirical TB treatment (assuming 70% patients complete a full 6-month and 30% of patients complete a 3-month course)	$634.91	calculated

Table S6: Costs and outcomes associated with unnecessary and empirical treatment for single, sequential and concurrent test strategies to diagnose TB in hospitalized patients with advanced HIV using Xpert MTB/RIF and LAM urine tests. Costs are expressed in 2018 $US with 95% CI in parentheses.

	**Single test strategies**		**Sequential test strategies**		**Concurrent testing strategy**
	**Xpert only**	**LAM only**	**LAM in Xpert-ve**	**Xpert in LAM-ve**	**Xpert+LAM**
**Total costs associated with unnecessary treatment (per 1000 patients screened)**					
Total cost	$148,653 (141435, 158911)	$174,487 (163090, 188544)	$187,235 (169068, 218473)	$186,228 (168745, 218566)	$186,645 (174487, 202601)
Costs incurred by false positives initiating treatment	$249,88 (15299, 38757)	$59,665 (44366, 78533)	$76,775 (54005, 108414)	$75,424 (52203, 108642)	$75,983 (59665, 97401)
Costs incurred by true negatives initiating treatment	$123,666 (120155, 126137)	$114,823 (118724, 110012)	$110,460 (110060, 115063)	$110,805 (109924, 116543)	$110,662 (105201, 114823)
**Outcomes (per 1000 patients screened)**					
Number of suspected TB patients who were empirically treated	218.9 (216.9, 220.0)	239.7 (238.6, 239)	194.9 (195.1, 191.9)	195.4 (191.4, 196)	195.2 (193.1, 196.2)
Number of culture negative patients who were initiated on TB treatment	228.3 (219.2, 241.2)	260.8 (246.4, 278.5)	276.8 (248.3, 312.8)	275.5 (247.3, 311.7)	276.1 (260.8, 296.1)
Number of missed TB cases (false negatives)	56.2 (42.5, 71.8)	137.3 (120.4, 153.3)	48.6 (36.4, 61.8)	48.8 (40.8, 53.7)	48.7 (35.8, 63.8)

FIGURES

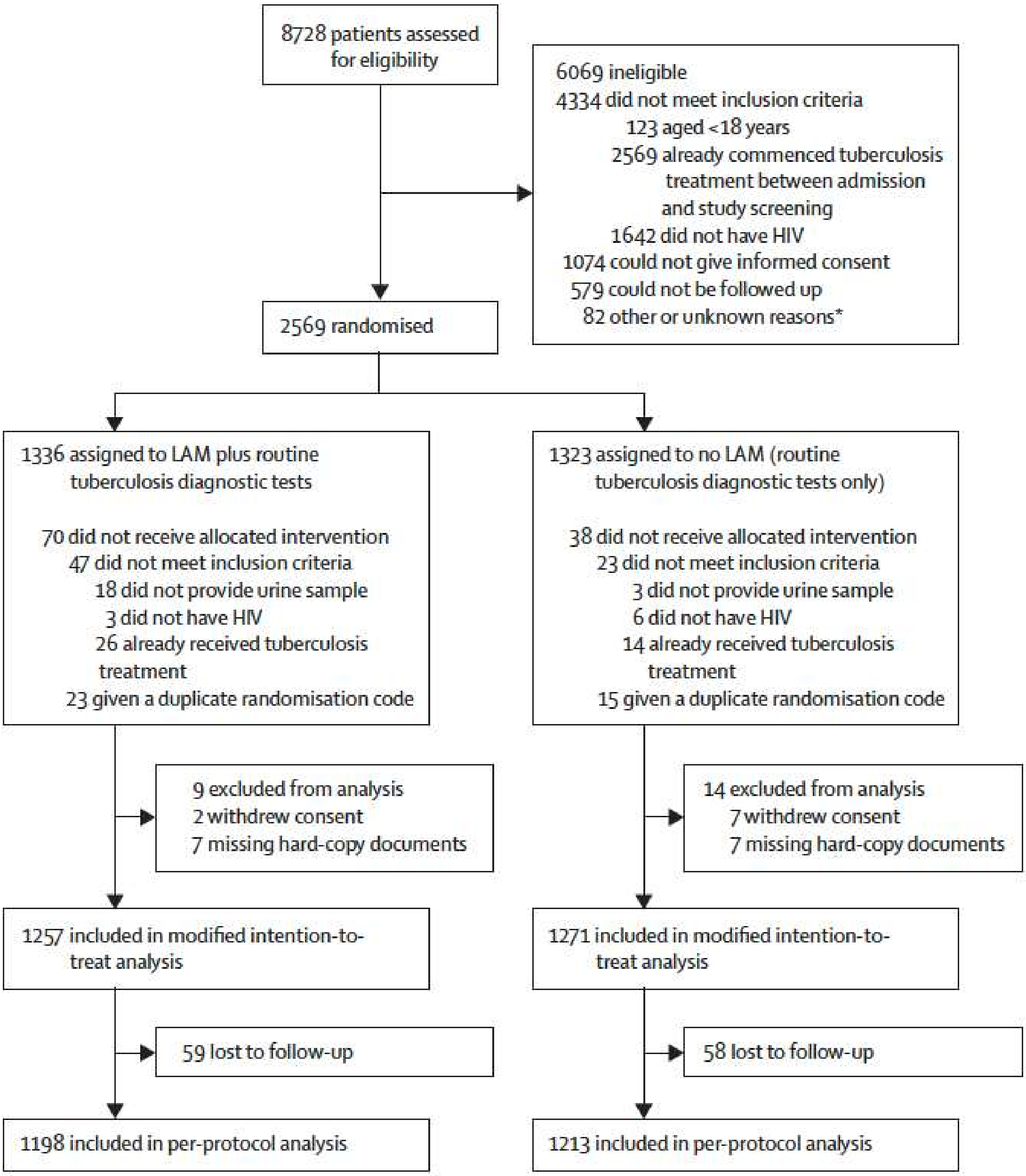

Figure S1: Study plan of the parent study (LAM RCT: [5])

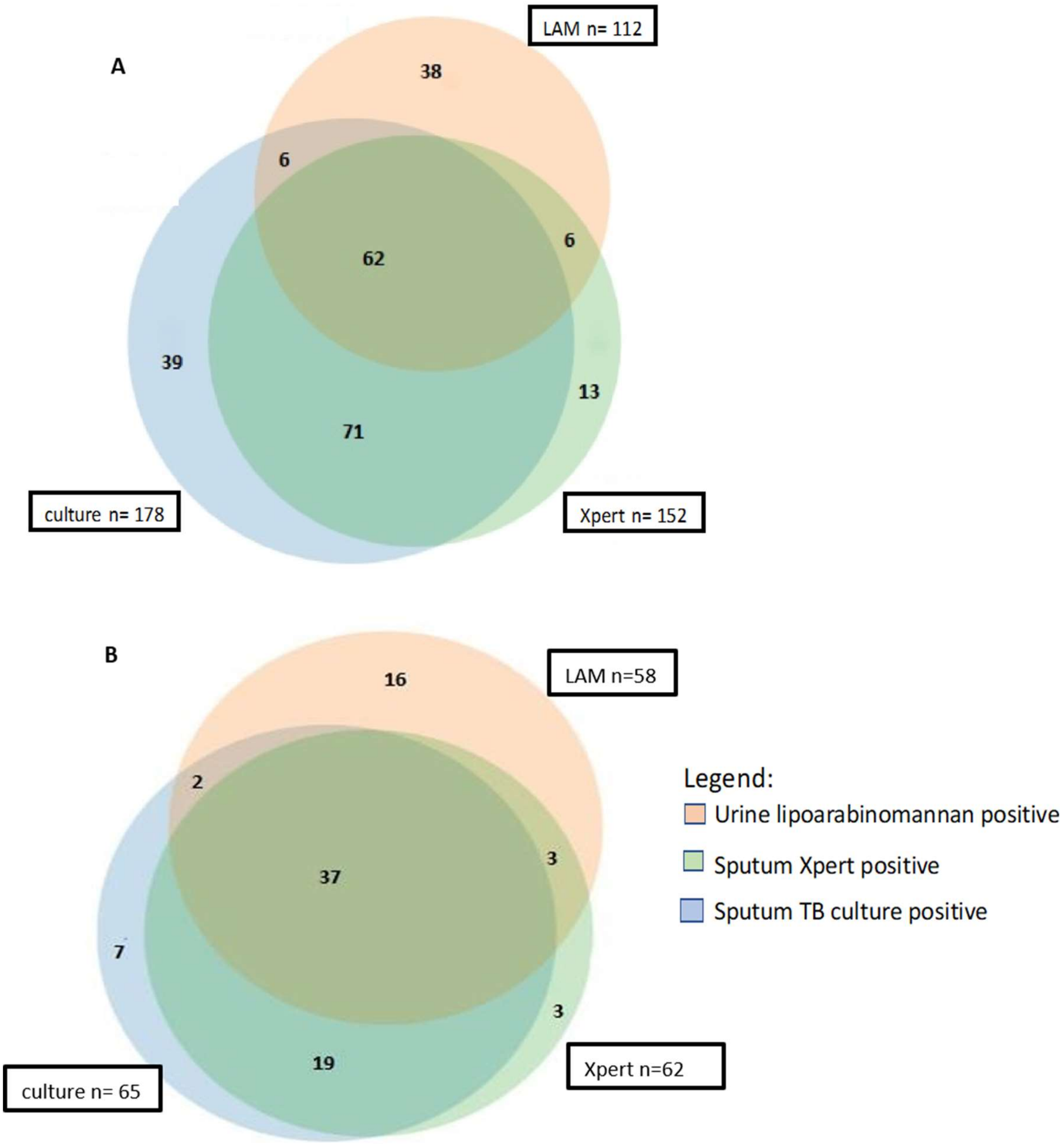

Figure S2: VENN Diagram demonstrating proportion of positive test results (sputum Xpert, sputum culture and urinary LAM) in (A) all patients (n=235) and (B) patients with CD4+ ≤ 50 cells/ul (n= 87).

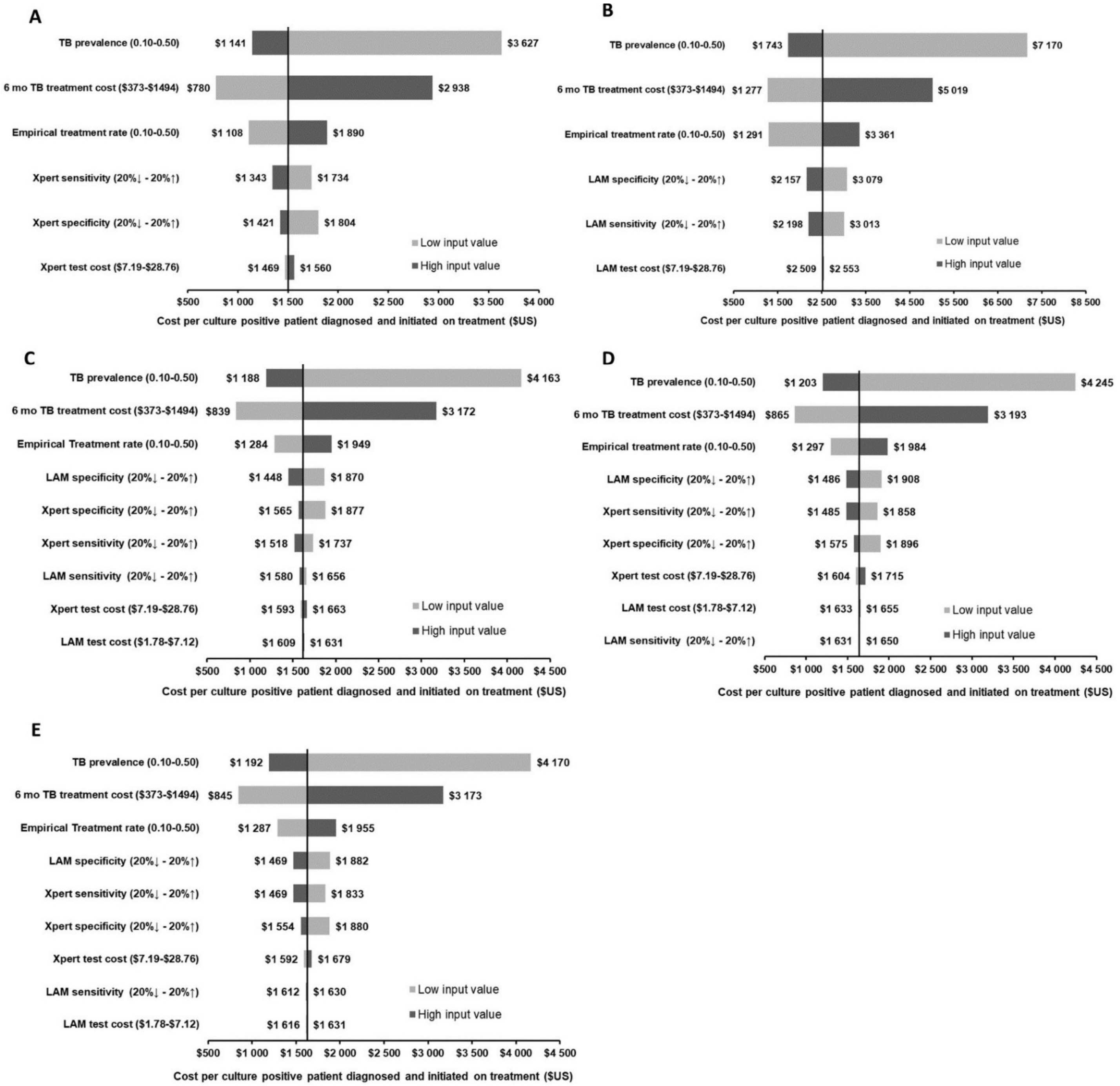

Figure S3: Univariate sensitivity analysis. Tornado diagrams outlining the effect of changing a single parameter in terms of the cost per culture positive case diagnosed and initiated on treatment for (A) Xpert only (B) LAM only (C) Xpert in LAM -ve patients and (D) LAM in Xpert -ve patients and (E) LAM+Xpert. Cost are expressed in 2018 $US

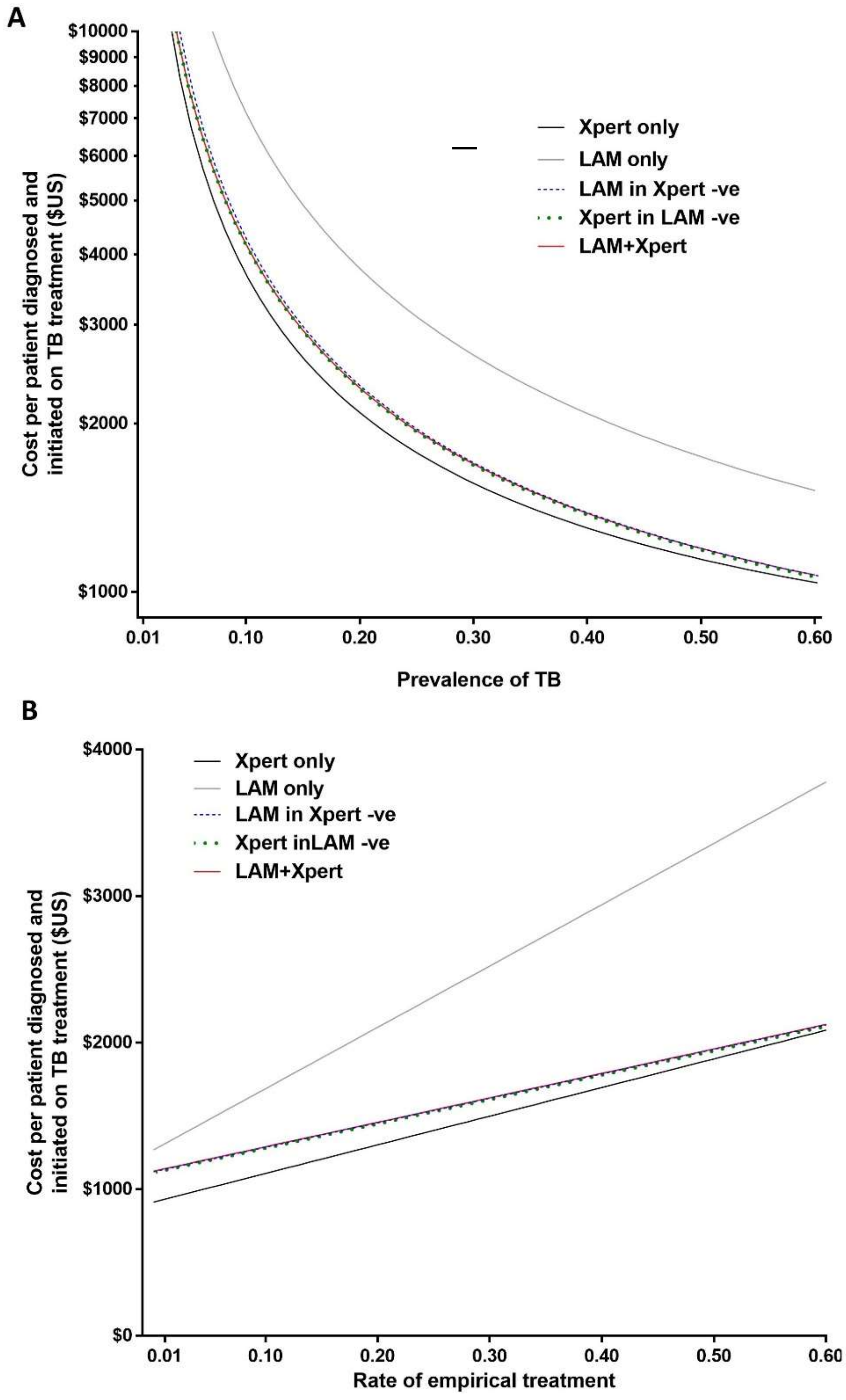

Figure S4. Effect of varying the (A) prevalence of TB and (B) rate of empirical treatment on the cost per culture positive case diagnosed and initiated on treatment.

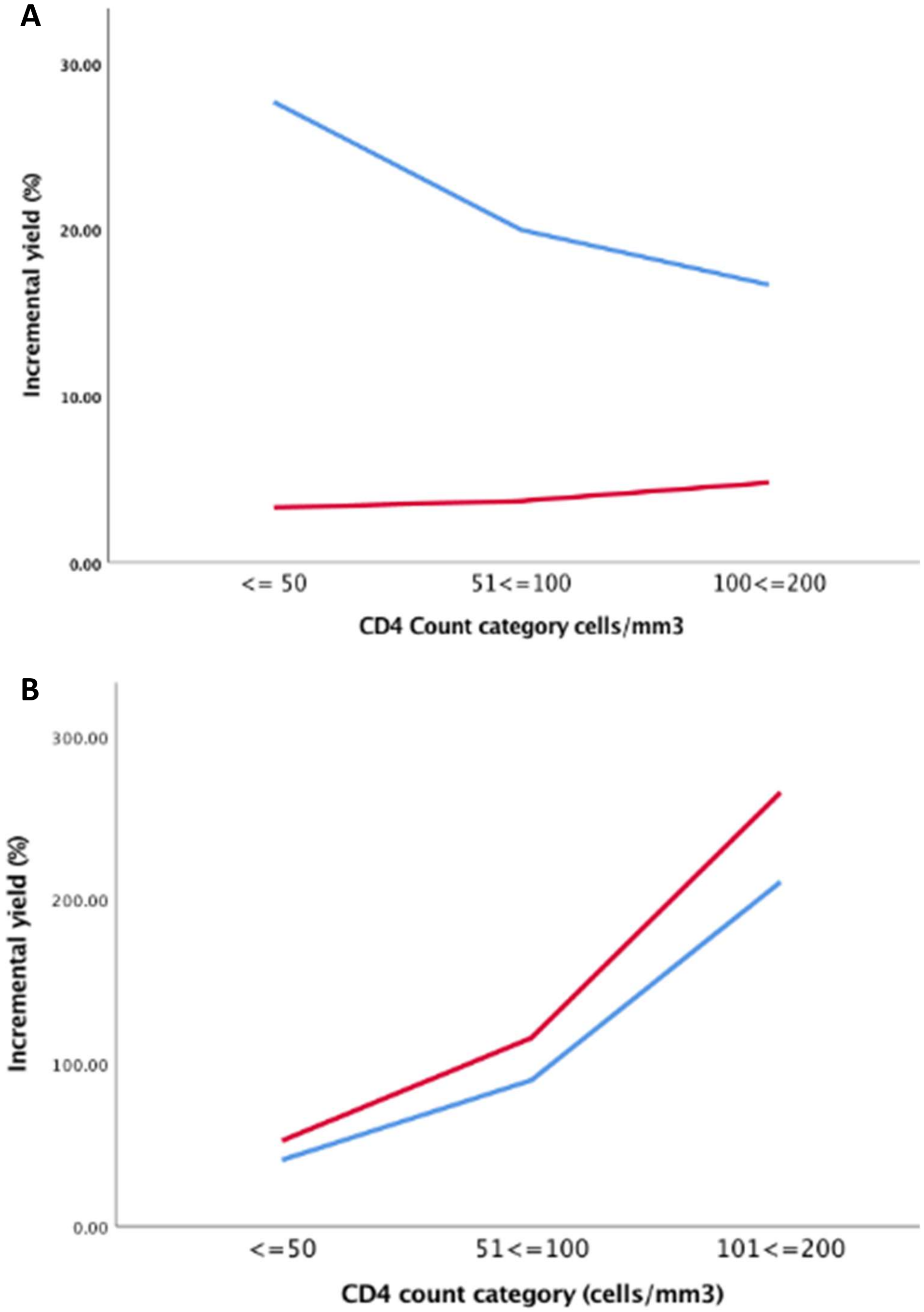

Figure S5. Incremental yield of (A) LAM (LAM positivity in Xpert-ve patients) and (B) Xpert (Xpert positivity in LAM-ve patients), stratified by CD4 count using any positive TB test (blue line) and sputum culture (red line) as a reference.
